# The Clinical Utility of Serum Biomarkers in the Differentiation and Prognostic Assessment of Viral Meningitis

**DOI:** 10.3390/pathogens15020234

**Published:** 2026-02-20

**Authors:** Georgiana Enache-Leonte, Andrei Vâță, Maria Ioana Onofrei, Mihnea Eudoxiu Hurmuzache, Gabriela Rusu Zota, Dan Trofin, Ioana Alina Harja-Alexa, Mihaela Cătălina Luca

**Affiliations:** 1 Department of Infectious Diseases (Internal Medicine II), Faculty of Medicine, University of Medicine and Pharmacy “Gr. T. Popa”, 700115 Iasi, Romania; georgiana.enache-leonte@umfiasi.ro (G.E.-L.); maria-ioana.hunea@umfiasi.ro (M.I.O.); mihnea.hurmuzache@umfiasi.ro (M.E.H.); ioana-alina.harja-alexa@umfiasi.ro (I.A.H.-A.); mihaela.luca@umfiasi.ro (M.C.L.); 2 St. Parascheva Clinical Hospital of Infectious Diseases, 700116 Iasi, Romania; 3 Department of Morpho-Functional Sciences II (Pharmacology, Clinical Pharmacology and Algeziology), Faculty of Medicine, Grigore T. Popa University of Medicine and Pharmacy of Iasi, 16 Universitatii Street, 700115 Iasi, Romania; rusu.i.gabriela@umfiasi.ro; 4 “Sf. Spiridon” Clinical Emergency Hospital, 700111 Iasi, Romania; 5 Department of Biomedical Sciences, Faculty of Medical Bioengineering, University of Medicine and Pharmacy “Gr. T. Popa”, 700454 Iasi, Romania; trofin.dan@umfiasi.ro

**Keywords:** viral meningitis, systemic immune–inflammation index, neutrophil-to-lymphocyte ratio, glutathione peroxidase-3, superoxide dismutase

## Abstract

*Background and Objectives*: Viral meningitis presents significant diagnostic challenges. The aim of this study was to identify predictive factors for meningitis etiology and clinical outcomes. *Methods*: This prospective, single center, comparative study enrolled patients meeting clinical, biological, and microbiological criteria for bacterial (BM) or viral (VM) meningitis. Serum inflammatory markers, namely the neutrophil-to-lymphocyte ratio (NLR), platelet-to-lymphocyte ratio (PLR), and systemic immune-inflammation index (SII), were quantified. In addition, the concentrations of oxidative stress biomarkers, glutathione peroxidase 3 (GPx3) and copper/zinc superoxide dismutase (Cu/Zn SOD), were also determined. Spearman correlation and logistic regression were used to evaluate associations between biomarkers and etiology, while receiver operating characteristic (ROC) curves assessed outcome correlations. *Results*: VM patients showed a significantly lower NLR (*p* = 0.007), PLR (*p* = 0.010), and SII (*p* = 0.016), with higher GPx3 (*p* < 0.0001) levels compared with BM patients. Cu/Zn SOD showed no significant difference (*p* = 0.442) between groups. Multivariate logistic regression identified the SII (OR = 1.015; 95% CI = 1.004–1.026) and GPx3 (OR = 0.847; 95% CI = 0.740–0.970) as etiology predictors. The SII was the strongest predictor of mortality in VM (AUC = 0.833). *Conclusions*: Serum markers of inflammation, including the systemic immune–inflammation index, along with antioxidant indicators such as GPx3, may serve as valuable additional tools for predicting meningitis etiology and forecasting patient outcomes.

## 1. Introduction

Viral meningitis (VM), often termed aseptic meningitis, represents a significant public health concern due to its increasing prevalence, diagnostic challenges, and potential long-term consequences [1]. Viral pathogens, including enteroviruses, herpes simplex viruses, and arboviruses, contribute to the rising burden of the disease across various age groups [1,2]. One notable trend in the etiology of viral meningitis is the rising incidence of enteroviral infections. Their proportion has increased significantly over recent decades, from 3% in earlier years (1968–1985) to 47% in the 2007–2011 period in the UK. This shift reflects the adoption of molecular diagnostic methods, which are more effective than traditional culture techniques [3].

The clinical presentation of viral meningitis frequently overlaps with that of bacterial meningitis, complicating initial diagnosis. Symptoms such as headache, fever, and neck stiffness can manifest in both types, making empirical antibiotic treatment common until a definitive diagnosis is established [4,5]. Despite being generally considered a self-limited benign illness, viral meningitis can lead to long-term complications, as many patients, even those with a mild course, may suffer from residual sequelae, significantly affecting their social and professional lives [6].

On the other hand, bacterial meningitis is an infectious emergency associated with a mortality rate of up to 30% and neurological sequelae in up to 50% of survivors [7]. Therefore, the prompt initiation of antibiotic therapy is essential in bacterial meningitis.

However, distinguishing viral from bacterial meningitis remains challenging, especially when cerebrospinal fluid (CSF) analysis shows atypical findings [8]. Current research emphasizes the need for sensitive and specific biomarkers to enhance diagnostic accuracy, particularly when conventional markers overlap or a lumbar puncture cannot be performed [9,10].

CSF analysis remains the gold standard for diagnosing meningitis. The diagnostic landscape for viral meningitis has evolved, with advancements, like the FilmArray Meningitis/Encephalitis panel, that streamline pathogen detection [11]. However, in circumstances where lumbar punctures are contraindicated or delayed, alternative biomarkers that can distinguish viral from bacterial meningitis are urgently needed.

An ideal biomarker for infections, e.g., leukocyte count, erythrocyte sedimentation rate (ESR), C-reactive protein (CRP), and interleukin-6 (IL-6), should enable rapid diagnosis, predict disease progression and prognosis, and inform therapeutic decision-making, including antibiotic administration [12].

Several biomarkers derived from differential counts have been studied and shown to be effective for diagnosing and predicting outcomes in various types of infections. The neutrophil-to-lymphocyte ratio (NLR), platelet-to-lymphocyte ratio (PLR), and systemic immune–inflammation index (SII) are significant non-invasive predictors used both for diagnosing and monitoring the evolution of infections or non-infectious disorders [13,14,15,16].

In addition, oxidative stress (OS) plays a significant role in the pathophysiology of viral meningitis, contributing to a complex interplay among viral infections, reactive oxygen species (ROS), and the central nervous system (CNS). Understanding these mechanisms is essential for developing therapeutic strategies aimed at mitigating oxidative damage. Viral infections are commonly associated with elevated OS due to increased ROS production. Elevated levels of oxidants, such as peroxynitrite, nitric oxide, and hydroxyl radicals, contribute to both viral pathogenesis and the modulation of host cellular responses and viral replication [17]. This excessive production of ROS leads to cellular damage, manifested as lipid peroxidation, protein oxidation, and DNA damage, thereby amplifying the virus’s pathogenic effects in neural tissues [18]. In viral meningitis, oxidative and nitrosative stress can significantly alter OS markers in patients. Both cerebrospinal fluid (CSF) and blood samples from patients with viral meningitis may exhibit pronounced OS profiles, suggesting that this condition triggers substantial oxidative damage [19]. Such findings underscore that the disruption of the oxidative balance reflects the disease’s severity and can contribute to the neuroinflammatory responses typically observed in viral meningitis.

The glutathione peroxidase (GPx) family plays a critical role in preserving cellular redox homeostasis and mitigating excessive reactive oxygen species (ROS) production, which is closely associated with the pathogenesis of numerous diseases, including viral infections [20]. Glutathione peroxidase-3 (GPx3) isoenzyme is an important biomarker for assessing the extent of inflammation and adverse outcomes in various systemic inflammatory diseases and cancers [21].

Cu/Zn superoxide dismutase (Cu/Zn SOD-1) is another essential antioxidant enzyme that has been investigated in the context of inflammatory disorders, chronic renal disease, and neurological conditions [22,23].

Therefore, this study aimed to evaluate the prognostic value of serum inflammatory markers (NLR, PLR, and SII) and antioxidant enzymes (GPX3 and Cu/Zn SOD) for diagnosis and disease progression in viral meningitis.

## 2. Materials and Methods

### 2.1. Study Design

We conducted a prospective, single-center, comparative, observational study of patients with community-acquired acute meningitis, admitted to the Clinical Hospital of Infectious Diseases “Sf. Parascheva” Iasi, Romania, between January 2020 and December 2024.

### 2.2. Study Population

The study cohort comprised 143 patients: 61 with viral meningitis and 82 with bacterial meningitis.

#### 2.2.1. Inclusion Criteria

This study included patients with confirmed or suspected acute community-acquired meningitis based on clinical manifestations (fever, headache, neck stiffness, altered mental status), CSF analysis and microbiological confirmation by culture, PCR, or antigen testing when available.

#### 2.2.2. Exclusion Criteria

Patients with other CNS infections (mycobacterial or fungal meningitis, encephalitis without meningitis, brain abscess, hospital-acquired meningitis, or chronic or recurrent meningitis) or non-infectious CNS pathological conditions (cerebral hemorrhage or infarction, malignancies, or autoimmune disorders), severe immunosuppression (e.g., advanced HIV, chemotherapy), or prior antibiotic treatment (within 48 h before admission) were excluded.

### 2.3. Data Collection

#### 2.3.1. Measurements at Admission

Patient assessments were conducted in accordance with standard medical practice. The following data were collected from the medical records of patients who gave consent for participation: demographic data (age, gender), medical and medication history, Charlson Comorbidity Index, and the history of CNS infections (symptoms at onset, duration). Neurological assessment at admission consisted of the calculation of the Glasgow Coma Scale (GCS), which was classified as severe (3 to 8 points), moderate (9 to 12 points) and mild (13 to 15 points) [24].

#### 2.3.2. Collection and Analysis of Samples

Within 30 min after admission and before the initiation of antibiotic therapy, blood samples were drawn, and a lumbar puncture was performed.

The laboratory data collected for this study included full blood count (absolute counts and percentages), biochemistry (liver enzymes, blood urea nitrogen, creatinine, blood glucose, CRP) and serum biomarkers for oxidative stress (GPx3 and Cu/Zn SOD). CSF analysis included microscopic cell counting, biochemistry (proteins, glucose, chloride), and a bacteriological assay. All tests were performed on the day of admission.

The complete blood count was attained using XS-1000i hematology analyzers (Sysmex, Norderstedt, Germany). The biochemical parameters defined above were measured using the Rx Imola (Randox, Antrim, UK). The BD Directigen^TM^ Meningitis Combo Test antigen kit (BD, Sparks, MD, USA) was used for the detection of bacterial antigens (*Neisseria meningitides* A, C, Y and W135, *Streptococcus pneumoniae*, Group B streptococcus *Escherichia coli* K1, and *Haemophilus influenzae* type b). The microbiological analysis of CSF included Gram staining and bacterial cultures. The PCR method using GeneXpert^®^ System (Cepheid, Sunnyvale, CA, USA) was applied for the identification of viral etiology.

Another blood sample (5 mL) was collected after admission for each included patient, before the initiation of therapy. After centrifugation, plasma samples were stored at −80 °C until analysis. A specific enzyme-linked immunosorbent assay using UT6500 (MRC, Holon, Israel) was performed to quantitatively analyze GPx3 and Cu/Zn SOD. The preparation of reagents and samples, as well as the assay, was performed according to the manufacturer’s instructions (BioVendor—Laboratorní medicína a.s., Brno, Czech Republic) [25,26]. The results were expressed as ng/mL.

In the case of suspected West Nile virus infection, serological samples (IgM antibodies) were sent for analysis to the Iasi Regional Public Health center.

#### 2.3.3. Outcome Variables

The following variables were calculated at admission: the NLR (neutrophil-to-lymphocyte ratio) in serum PLR (platelet-to-lymphocyte ratio) and SII (systemic immune–inflammation index) = (neutrophils × platelets)/lymphocytes.

The bacterial meningitis score (BMS) was calculated for each patient. The BMS, recommended by pediatric academic societies, is a validated tool for estimating the risk of bacterial meningitis. This score comprises five variables: serum (absolute neutrophil count ≥ 10,000 cells/µL) and CSF (positive Gram stain, absolute neutrophil count ≥ 1000 cells/µL, protein ≥ 80 mg/dL) and the presence of seizures [27]. A BMS score of 2.5 or higher was used as the cut-off for bacterial meningitis [28].

#### 2.3.4. Data Collection During Hospitalization and Discharge

During hospitalization, data on treatment, the duration of hospitalization, and clinical course were extracted from patients’ files.

At discharge, the disability level was assessed using the Modified Rankin Scale (mRS), a 6-point scale from 0 (no symptoms) to 6 (death). Outcomes were categorized into three groups based on mRS scores: “good” (scores 0–2), “intermediate” (scores 3–4), and “poor” (scores 5–6) [29].

### 2.4. Statistical Analysis

Normally distributed data were presented as means ± standard deviations and coefficients of variation (CV%), and non-normally distributed data were reported as medians and ranges. Qualitative data was expressed as percentages. The *t*-test or chi-squared test was performed for the single-factor analysis of count data, and the Mann–Whitney test was performed for the single-factor analysis of non-normally distributed data.

Spearman’s correlation analysis was used to assess relationships among variables, and multiple logistic regression was used to screen predictors of bacterial or viral etiology. Age, comorbidities, and neurological impairment (based on GCS score) were additional covariates tested for association with meningitis outcomes. The impact of serum inflammatory markers and antioxidants on the outcome of viral and bacterial meningitis was analyzed using receiver operating characteristic (ROC) curves. A *p*-value below 0.05 was considered statistically significant in all the statistical tests. Analyses were performed using XLSTAT software (Lumivero, 2024; XLSTAT statistical and data analysis solution, Paris, France).

## 3. Results

### 3.1. Characteristics of Participants

**Figure 1:**
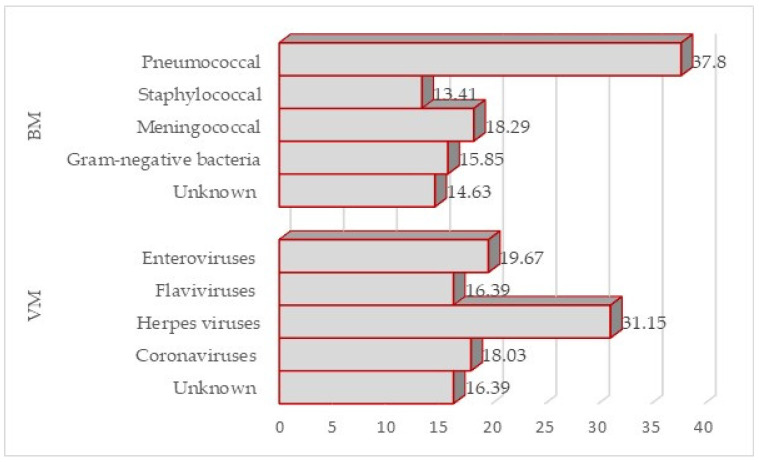
Etiology of BM and VM in study patients (*n* = 143).

Of 220 patients, complete datasets were obtained from 143 patients, of whom 57.34% (*n* = 82) had BM, and 42.66% (*n* = 61) had VM (Figure 1).

The demographic, medical and biological characteristics of included patients are presented in Table 1.

In the VM group, herpesviruses were the predominant cause at 31.15%, followed by enteroviruses at 19.67% and West Nile virus at 16.39%, with SARS-CoV-2 accounting for 18.03% of infections.

The VM group had a median age of 67 years (range 23–88 years), with male patients significantly outnumbering female patients (*p* = 0.039).

The microbiological characterization of the BM group revealed heterogeneous pathogen distribution: pneumococcal etiology predominated at 37.8%, whereas meningococcal and Gram-negative bacterial isolates accounted for 18.29% and 15.85% of cases, respectively.

In 16.39% (*n* = 10) of VM cases and 14.63% (*n* = 12) of BM cases, the etiology was not established, even though none of the patients reported antibiotic use prior to admission.

The median age in the BM group was 55 years (range 22–82 years), with no significant gender distribution (*p* = 0.717).

Comparing the two groups, it was noted that the age of patients with VM was statistically significantly higher than that of patients with BM (*p* = 0.006) (Table 1). In the multivariate logistic regression analysis, age was not a predictor for the type of meningitis (OR = 0.949; 95% CI = 0.91–1.99) or for mortality (OR = 0.990; 95% CI = 0.957–1.025).

While the Charlson index showed no significant difference between groups (*p* = 0.906), patients with BM had a significantly higher incidence of liver disease than those with VM (*p* < 0.0001) (Table 1). In our study, multivariate logistic regression showed that liver disorders were not associated with an unfavorable prognosis (OR = 0.494; 95% CI = 0.146–1.67).

An analysis of the BMS scoring system revealed significantly lower median scores in patients with VM (1.9; range 1.6–2.20) than those with BM (2.65; range 2.52–4.0).

### 3.2. Serum Inflammatory Biomarkers in Bacterial and Viral Meningitis

Compared with BM patients, those with VM exhibited significantly lower blood leukocyte counts (median 10.92 vs. 17.09; *p* < 0.0001), lower neutrophil counts (median 6.6 vs. 76.95; *p* < 0.0001), and higher lymphocyte counts (median 86.2 vs. 14.95; *p* < 0.0001). No significant differences were observed in platelet counts between groups (median 282.00 vs. 191.00, *p* = 0.244). Additionally, CRP values were significantly lower in viral meningitis compared to bacterial meningitis (median 16.2 vs. 139.08, *p* < 0.0001) (Table 1).

All calculated inflammatory biomarkers (SII, NLR, PLR) were significantly higher in BM compared to VM. The medians were 5.94 in VM vs. 13.35 in BM (*p* = 0.007) for the NLR, 164.9 in VM vs. 264.3 in BM (*p* = 0.010) for the PLR, and 1233.54 in VM vs. 2308.93 in BM for the SII (*p* = 0.016) (Table 2).

### 3.3. Serum Oxidative Stress Markers in Viral and Bacterial Meningitis

In our study, GPx3 serum levels (ng/mL) were significantly lower in patients with BM than in those with VM (3.7 ± 0.81 vs. 7.02 ± 1.01; *p* < 0.0001).

**Figure 2:**
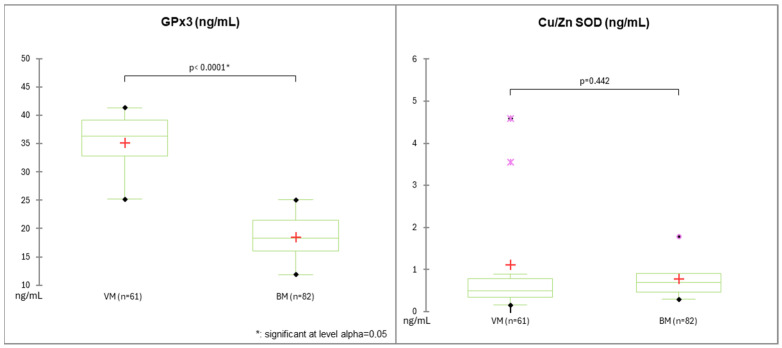
Boxplots showing the difference between markers of oxidative stress in bacterial and viral meningitis. GPx3, glutathione peroxidase-3; CU/ZN SOD, Cu/Zn superoxide dismutase; VM, viral meningitis; BM, bacterial meningitis. *: significant at level alpha = 0.05.

In contrast, no significant differences in Cu/Zn SOD levels (ng/mL) were found between patients with BM and VM (0.15 ± 0.09 vs. 0.22 ± 0.18, *p* = 0.442) (Figure 2).

### 3.4. Diagnostic Value of NLR, PLR, SII, GPx3 and Cu/Zn SOD for BM and VM

The Spearman correlation test showed a strong relationship between the SII (Spearman coefficient 0.861, *p* < 0.0001), Gpx3 (Spearman coefficient 0.864, *p* < 0.0001), and the BMS.

Among BMS values greater than or equal to 2.5 relevant for BM, the strongest significant correlation was between the BMS and SII (Spearman coefficient 0.864), followed by the NLR (Spearman coefficient 0.856) and CRP (Spearman coefficient 0.782). A very strong negative correlation was observed between the BMS value and GPx3 (Spearman’s coefficient = −0.861). Cu/Zn SOD was very weakly correlated with the BMS (Table 2).

Considering values less than 2.5 specific to VM, the strongest significant correlation was between the BMS and SII (Spearman coefficient 0.841), followed by CRP (Spearman coefficient 0.798).

A very strong negative correlation was observed between the BMS value and GPx3 (Spearman’s coefficient = −0.854, <0.0001). In this group, only a weak correlation was found between the NLR and BMS (Table 3).

However, the multivariate logistic regression analysis showed that among the inflammatory markers studied, only the SII (OR = 1.015; 95% CI = 1.004–1.026) and GPx3 (OR = 0.847; 95% CI = 0.740–0.970) have the potential to predict viral or bacterial etiology in meningitis (Table 4).

To validate these findings, we generated an ROC curve for predicting viral meningitis using the BMS (Table 5).

Among the biomarkers evaluated, the NLR (cut-off 7.39, AUC = 0.875, sensitivity 86%, specificity 75%), PLR (cut-off 204.95, AUC = 0.852, sensitivity 86.4%, specificity 71.9%) and SII (cut-off 1712.70, AUC = 0.741, sensitivity 71.4%, specificity 73.3%) showed the highest diagnostic accuracy for viral meningitis (Table 5).

GPx3 (cut-off 4.26 ng/mL, AUC = 0.67, sensitivity 88.9%, specificity 50%) and Cu/Zn SOD (cut-off 0.08, AUC = 0.61, sensitivity 90.5%, specificity 43.3%) had moderate predictability (Table 5).

### 3.5. Prognostic Value of NLR, PLR, SII, GPx3 and Cu/Zn SOD for Outcomes in BM and VM

An analysis of mRS scores revealed that patients with viral meningitis had significantly better clinical outcomes compared to those with bacterial meningitis (57.38% vs. 31.71%, *p* = 0.003). While overall poor outcomes did not differ significantly between groups, mortality rates were significantly lower in viral meningitis than in bacterial meningitis (11.47% vs. 25.61%, *p* = 0.044).

Spearman correlation analysis revealed strong associations between mortality and both comorbidity burden (Charlson index; Spearman coefficient = 0.556, *p* = 0.003) and cognitive impairment (GCS score; Spearman coefficient =−0.429, *p* = 0.026) in patients with either form of meningitis. The initial serum concentrations of GPx3 and Cu/Zn SOD at hospital admission were not associated with mortality outcomes in either BM or VM (Table 6).

To assess the relationship between the analyzed biomarkers and mortality, ROC curves were generated for both the viral meningitis and bacterial meningitis groups.

**Figure 3:**
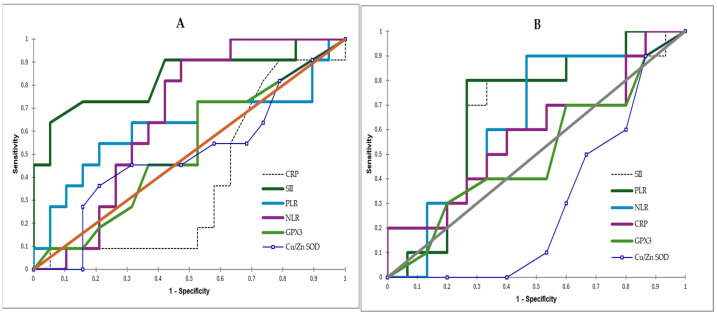
ROC curves of scoring system with risk factors for negative outcome in VM (**A**) and BM (**B**). CRP, C-reactive protein; NLR, neutrophil-to-lymphocyte ratio; PLR, platelet-to-lymphocyte ratio; SII, systemic immune–inflammation index; GPx3, glutathione peroxidase 3; Cu/Zn SOD, Cu/Zn superoxide dismutase.

In VM, the SII emerged as the strongest predictor of mortality, achieving an AUC of 0.7735 (sensitivity: 70%; specificity: 76.5%; cut-off: 1365.65). The PLR (AUC = 0.718; sensitivity: 50%; specificity: 88.2%; cut-off: 287.83) and NLR (AUC = 0.641; sensitivity: 60%; specificity: 76.5%; cut-off: 27.63) demonstrated weaker predictive performance. Both GPx3 (AUC = 0.44; sensitivity: 20%; specificity: 88%) and Cu/Zn SOD (AUC = 0.515; sensitivity: 40%; specificity: 76.5%) showed no predictive capacity for mortality in viral meningitis (Figure 3A).

Comparable findings were observed in patients with BM. The SII remained the strongest mortality predictor (AUC = 0.775; sensitivity: 64.7%; specificity: 82.7%; cut-off: 1863.53), with the PLR (AUC = 0.735; sensitivity: 70.6%; specificity: 66.7%; cut-off: 308.0) and NLR (AUC = 0.706; sensitivity: 88.2%; specificity: 66.7%; cut-off: 27.63) showing moderate predictive capacity. GPx3 (AUC = 0.42; sensitivity: 35.3%; specificity: 66.7%) and Cu/Zn SOD (AUC = 0.525; sensitivity: 41.2%; specificity: 16.7%) similarly showed no significant association with mortality (Figure 3B).

## 4. Discussion

Rapidly distinguishing between viral and bacterial meningitis remains clinically challenging due to overlapping symptoms—fever, headache, meningism, and altered mental status—particularly when atypical cerebrospinal fluid findings, such as neutrophilic pleocytosis, obscure the diagnostic picture [11,30]. While polymerase chain reaction technology has revolutionized viral pathogen detection, its limited global accessibility, imperfect sensitivity, and variable CSF cellular profiles complicate timely diagnosis [31,32,33]. Infrastructure and economic constraints in resource-limited settings perpetuate reliance on conventional diagnostics, delaying appropriate antimicrobial stewardship [30,34].

We evaluated various non-invasive biomarkers for diagnosis and outcomes in bacterial and viral meningitis among 143 patients hospitalized at our hospital between January 2020 and December 2024.

The etiological distribution of meningitis cases in our study was consistent with prior reports. *Streptococcus pneumoniae* was the predominant pathogen in bacterial meningitis, whereas varicella zoster virus was the most frequently identified causative agent in viral meningitis [35].

Traditional biomarkers, including procalcitonin and CSF lactate, exhibit inconsistent discriminatory performance, underscoring the need for novel complementary parameters [36].

The bacterial meningitis score (BMS) using cut-offs of ≥2 in pediatric populations and ≥2.5 in adults provides improved diagnostic accuracy, though the integration of additional hematological indices may enhance etiological differentiation [24,25]. Obreja et al. (2022) noted that the blood levels of S100 calcium-binding protein B could differentiate BM from VM (cut-off value of 36.24 ng/mL, AUC 0.652, sensitivity 65%, specificity 57.14%) [37].

In our study, we analyzed three parameters derived from a full blood count: the neutrophil-to-lymphocyte ratio (NLR), platelet-to-lymphocyte ratio (PLR), and systemic immune–inflammation index (SII).

The NLR and PLR could play a role as significant biomarkers for diagnosis and mortality in various infectious (e.g., sepsis, COVID-19, meningitis) or non-infectious chronic conditions (e.g., heart failure, atherosclerotic cardiovascular disease, cancer, and chronic inflammatory diseases) [13,38,39,40].

Consistent with the existing literature, our cohort demonstrated a robust correlation between the NLR and BMS in bacterial meningitis (Spearman’s rho = 0.856, *p* < 0.0001), whereas in viral meningitis cases, the correlation was moderate but significant (Spearman’s rho = 0.463; *p* = 0.006). Our study identified an NLR cut-off value of 7.391 (AUC = 0.875; sensitivity: 75%; specificity: 84%), which aligns closely with previously published findings. In a comprehensive analysis of 4339 acute meningitis cases, an NLR threshold of 8 (AUC = 0.677; sensitivity: 46.4%; specificity: 83%) was identified to discriminate between bacterial and viral meningitis [41]. Another investigation of 79 meningitis patients established an optimal NLR cut-off of 5.57 (AUC: 0.756; sensitivity: 79.17%; specificity: 61.29%) [42]. Holub et al. (2012) reported an NLR cut-off of 6.2 (AUC = 0.971; sensitivity = 91%; specificity = 0.96%) for distinguishing systemic bacterial from viral infections [43]. Similar NLR values have been observed in other severe systemic infections, including COVID-19 [44].

Our analysis determined a PLR cut-off value of 204.950 (AUC: 0.852; sensitivity: 71.9%; specificity: 75.6%), which is comparable to the findings of Kazancioglu et al. (2023), who reported a PLR threshold above 196 (AUC: 0.661; sensitivity: 60.42%; specificity: 70.97%) [42]. For acute herpes zoster meningitis specifically, PLR showed an AUC of 0.720 in predicting VZV meningitis, with an optimal cut-off yielding 58.1% sensitivity and 82.8% specificity [45]. The PLR values in our study were consistent with those reported in other severe infections. For instance, COVID-19 patients exhibited PLR values ranging from 153.6 in mild/asymptomatic disease to 257.77 in severe/critical disease [44].

The systemic immune–inflammation index (SII), a composite marker derived from routine blood counts, has shown preliminary utility in differentiating meningitis from encephalitis in emergency department settings, though its prognostic value for predicting mortality or neurological complications remains unestablished [46]. In our analysis, the SII was found to be a potential predictor for the diagnosis of bacterial meningitis and a predictor for mortality in viral meningitis (AUC = 0.7735; sensitivity: 70%; specificity: 76.5%) and bacterial meningitis (AUC = 0.775; sensitivity: 64.7%; specificity: 82.7%).

Beyond serum inflammatory biomarkers, our study also assessed antioxidant agents, specifically glutathione peroxidase 3 (GPx3) and Cu/Zn superoxide dismutase (Cu/Zn SOD).

The pathogenesis of viral meningitis encompasses complex oxidative stress pathways whereby reduced antioxidant defenses enable reactive oxygen species accumulation and resultant neurotoxic damage [47]. In patients with viral meningitis, elevated cerebrospinal fluid matrix metalloproteinase-9 levels are associated with oxidative injury, whereas nitric oxide exhibits both pro-oxidant and neuroprotective functions, depending on the physiological milieu [48,49].

Understanding meningitis pathophysiology requires examining how bacterial and viral pathogens disrupt glutathione homeostasis through fundamentally divergent mechanisms. In bacterial meningitis, a hyperinflammatory cascade ensues in which the host immune response becomes pathologically self-damaging, depleting glutathione reserves as oxidative stress intensifies. Critical glutathione depletion triggers ferroptosis pathways, propagating cellular injury from the central nervous system to peripheral organs such as the liver, thereby converting a localized infection into systemic pathology [50]. The evolving GSH-to-GSSG ratio functions as a biochemical indicator of sepsis severity, reflecting both oxidative stress magnitude and diminishing antioxidant capacity [51]. In viral meningitis, this metabolic manipulation results in measurable reductions in glutathione levels in cerebrospinal fluid and neural tissue [52].

GPx3, a member of the glutathione peroxidase family, has been investigated as a predictive biomarker across various pathological conditions. Among lung cancer patients undergoing surgery, postoperative alterations in serum GPx3 concentrations may indicate a higher risk of recurrence [53]. A study involving 36 critically ill sepsis/septic shock patients demonstrated that GPx3 levels effectively predicted disease severity based on systemic inflammatory response (SIRS) criteria. However, they showed no predictive value for mortality or multiple organ dysfunction. The optimal cut-off value was 0.5 U/mL (AUC = 0.921, 95% CI 0.844–0.999) [54].

The bacterial meningitis score served as our primary instrument for distinguishing between bacterial and viral meningitis. While originally developed for pediatric populations, this score remains applicable to adult patients. GPx3 showed a strong negative correlation with this score (Spearman’s coefficient = −0.854, *p* < 0.0001). Multivariate logistic regression further supported its diagnostic value (OR: 0.847; 95% CI: 0.740–0.970). The optimal cut-off value was 4.27 ng/mL (AUC = 0.667). However, our study found no predictive association between GPx3 and mortality in either bacterial or viral meningitis.

Superoxide dismutase regulates inflammatory responses by eliminating superoxide radicals (O_2_^−^), thereby blocking their transformation into highly reactive molecules such as hydrogen peroxide (H_2_O_2_) and hydroxyl radicals (·OH) that function as signaling intermediates in inflammatory cascades. By interfering with these processes, SOD inhibits redox-dependent pathways, including NF-κB and MAPK activation, leading to decreased proinflammatory cytokine release (such as TNF-α and IL-6) and facilitating macrophage phenotype transition from M1 to M2. During meningeal infections, pathogen-derived SOD counteracts immune cell-generated reactive oxygen species, whereas endogenous SOD mitigates inflammatory overactivation to minimize collateral tissue injury [55].

A Turkish study found elevated SOD levels in pediatric patients with bacterial meningitis and encephalitis compared with healthy controls. The mean SOD values reached 23.32 U/mL in bacterial meningitis cases, 29.21 U/mL in encephalitis patients, and 9.58 U/mL in control subjects [56].

Our study yielded results comparable to previously reported findings, though direct comparisons remain challenging due to differences in measurement units across studies. In our patient population, Cu/Zn SOD levels failed to differentiate among meningitis etiologies or predict clinical outcomes.

Antioxidants represent valuable biomarkers in infectious diseases by indicating oxidative stress imbalances, whereby pathogen invasion stimulates excessive reactive oxygen species (ROS) production, consequently depleting antioxidant defenses. During severe infections, the concentrations of enzymatic antioxidants such as SOD, catalase, and GPx, as well as nonenzymatic antioxidants such as GSH, frequently decline, thereby facilitating diagnostic assessment and severity stratification. Bacterial infections generally provoke more pronounced oxidative responses compared to viral infections, offering potential for differential diagnosis [57]. Furthermore, understanding the interplay with antioxidants has emerged as valuable for identifying potential therapeutic targets. Various antioxidants, including melatonin, selenium, zinc, copper, omega-3 fatty acids, curcumin, vitamin E, and N-acetylcysteine, elevate glutathione peroxidase (GPx) and SOD levels, potentially offering additional therapeutic benefits [55,58].

Substantial efforts are underway to achieve the rapid, accurate diagnosis of meningitis while simultaneously examining viral meningitis incidence across adult and pediatric populations. Although prompt diagnosis based on CSF analysis and treatment reduces mortality and neurological sequelae, delays may occur due to atypical disease presentation, hesitation to perform lumbar puncture, or the limited sensitivity of microbiological diagnostic methods [59]. In such cases, emerging evidence suggests that markers of systemic inflammation, including the neutrophil-to-lymphocyte ratio (NLR) and, to a lesser extent, the platelet-to-lymphocyte ratio (PLR) and the systemic immune–inflammation index (SII), may help distinguish bacterial from viral meningitis and predict adverse outcomes, such as mortality and neurological complications [46].

Limitations of this study. The sample size was limited, primarily due to the COVID-19 pandemic, which significantly reduced patient admissions to our institution, designated as a first-line COVID-19 treatment facility.

The etiological agent remained unidentified in a subset of cases in both groups. Because this observational study reflected real-world clinical practice, treating physicians had no discretion over PCR use, particularly during pandemic periods. However, patients were classified based on comprehensive clinical, laboratory, and cerebrospinal fluid criteria rather than microbiological confirmation alone, and prior antibiotic administration was an exclusion criterion. Additionally, the biomarkers studied were only quantified in blood, not in cerebrospinal fluid.

Further studies involving larger patient cohorts with viral or bacterial meningitis are needed to develop comprehensive, user-friendly algorithms that incorporate multiple systemic parameters and could serve as a diagnostic and prognostic tool for meningitis. However, such algorithms cannot replace the CSF analysis. Furthermore, when the etiological agent remains unidentified, expanded access to PCR-based diagnostic methods is essential to improve diagnostic accuracy.

## 5. Conclusions

Identifying new biomarkers with robust predictive capacity or developing enhanced diagnostic algorithms is critical for achieving greater diagnostic accuracy and ensuring the prompt administration of appropriate therapy in viral meningitis.

Serum markers of inflammation, including the systemic immune–inflammation index, along with antioxidant indicators such as GPx3, may serve as valuable additional tools for predicting meningitis etiology and forecasting patient outcomes.

## Figures and Tables

**Table 1:** Patients’ characteristics at admission by group (*n* = 143).

	VM (*n* = 61)			BM (*n* = 82)			
Parameter	Median	Minimum	Maximum	Median	Minimum	Maximum	*p*-Value
Demographics							
Age (years)	67	23	88	55	22	82	0.006
Gender (%)							
Female	52.45 (*n* = 32)			41.46 (*n* = 48)			0.580
Male	47.54 (*n* = 29)			58.53 (*n* = 34)			0.579
Comorbidities (%)							
Cardiovascular	40.98 (*n* = 25)			39.02(*n* = 32)			0.949
Respiratory	19.67 (*n* = 12)			26.83 (*n* = 22)			0.417
Liver diseases	37.7 (*n* = 23)			7.31 (*n* = 6)			<0.0001
Oncologic	8.19 (*n* = 5)			14.63 (*n* = 12)			0.422
Diabetes	21.31 (*n* = 13)			18.29 (*n* = 15)			0.814
	VM (*n* = 61)			BM (*n* = 82)			
	Median	Minimum	Maximum	Median	Minimum	Maximum	*p*-Value
Clinical							
Charlson index	2	0	8	3	0	8	0.906
GCS score	10	7	14	9	7	13	0.642
BMS	1.9	1.6	2.20	2.65	2.52	4	<0.0001
Laboratory							
Serum							
CRP (mg/dL)	16.20	0.10	107.95	139.08	2.37	634.72	<0.0001
WBC (× 10^3^/mmc)	10.92	0.15	25.27	17.09	10.23	35.33	<0.0001
Neutrophils (%)	6.60	1.20	48.80	76.95	42.10	92.30	0.003
Lymphocytes (%)	86.20	36.50	96.20	14.95	5.10	47.40	0.004
Platelets (× 10^3^/mmc)	282.00	81.00	482.00	191.00	71.00	998.00	0.244
CSF							
Cells	132.0	10	2460	2450	10	14,000	<0.0001
Neutrophils (%)	20.5	2	67	93	68	98	<0.0001
Lymphocytes (%)	79	33	98	6	2	27	<0.0001
Protein	0.79	0.21	1.4	3.71	0.86	154.4	0.003
Glucose	59	43	87	50	1	140	0.078
Outcome (%)							
Good	57.38 (*n* = 35)			31.71 (*n* = 30)			0.003
Intermediate	24.59 (*n* = 15)			40.24 (*n* = 34)			0.066
Poor	18.02 (*n* = 11)			28.05 (*n* = 23)			0.270
Hospitalization (days)	14	3	30	20	1	36	0.219

BMS, bacterial meningitis score; GCS, Glasgow Coma Scale; CSF, cerebrospinal fluid.

**Table 2:** Serum biomarkers (NLR, PLR and SII).

	VM (*n* = 61)			BM (*n* = 82)			
Biomarker	Median	Minimum	Maximum	Median	Minimum	Maximum	*p*-Value
NLR	5.94	0.89	17.55	13.35	0.75	78.5	0.007
PLR	164.92	21.30	595.42	264.32	192.1	1008.4	0.01
SII	1233.54	89.71	5257.25	2308.93	69.99	20,156.3	0.016

NLR, neutrophil-to-lymphocyte ratio; PLR, platelet-to-lymphocyte ratio; SII, systemic immune–inflammation index.

**Table 3:** Spearman correlation (SC) showing the correlation between the analyzed variables and BMS.

BMS	CRP		NLR		PLR		SII		GPx3		Cu/Zn SOD	
	SC	*p*-Value	SC	*p*-Value	SC	*p*-Value	SC	*p*-Value	SC	*p*-Value	SC	*p*-Value
≥2.5	0.782	<0.0001	0.856	<0.0001	0.168	0.219	0.864	<0.0001	−0.861	<0.0001	−0.069	0.219
<2.5	0.798	<0.0001	0.463	0.006	0.152	0.388	0.841	<0.0001	−0.854	<0.0001	−0.195	0.268

SC, Spearman coefficient; BMS, bacterial meningitis score; NLR, neutrophil-to-lymphocyte ratio; PLR, platelet-to-lymphocyte ratio; SII, systemic immune–inflammation index; CRP, C-reactive protein.

**Table 4:** Analysis results of logistic regression.

Biomarker	Wald Chi-Square	Pr > Chi^2^	Odds Ratio	Odds Ratio Lower Bound (95%)	Odds Ratio Upper Bound (95%)
CRP	2.549	0.110	0.954	0.900	1.011
SII	7.420	0.006	1.015	1.004	1.026
PLR	0.044	0.834	0.997	0.969	1.025
NLR	5.864	0.015	0.823	0.703	0.964
GPx3	5.731	0.017	0.847	0.740	0.970
Cu/Zn SOD	0.396	0.529	1.352	0.529	3.459

CRP, C-reactive protein; NLR, neutrophil-to-lymphocyte ratio; PLR, platelet-to-lymphocyte ratio; SII, systemic immune–inflammation index; GPx3, glutathione peroxidase 3; Cu/Zn SOD, Cu/Zn superoxide dismutase.

**Table 5:** Cut-off values and AUCs for NLR, PLR, SII, GPx3 and Cu/Zn SOD.

Biomarker	Cut-Off	AUC	Sensitivity	Specificity	PPV	NPV
SII	1712.701	0.741	0.714	0.733	0.728	0.720
NLR	7.391	0.875	0.864	0.750	0.846	0.670
PLR	204.950	0.852	0.864	0.719	0.754	0.841
GPx3	4.26	0.667	0.889	0.500	0.640	0.818
Cu/Zn SOD	0.08	0.613	0.905	0.433	0.615	0.820

AUC, area under the curve; PPV, positive predictive value; NPV, negative predictive value.

**Table 6:** Spearman correlation (SC) showing the correlation between the analyzed variables and mortality.

Variable	VM		BM	
	SC	*p*-Value	SC	*p*-Value
NLR	0.179	0.371	0.208	0.376
PLR	0.386	0.048	0.381	0.098
SII	0.504	0.008	0.414	0.071
GPx3	−0.025	0.904	−0.139	0.559
Cu/Zn SOD	0.003	0.990	0.193	0.414
Age	0.245	0.218	0.270	0.249
GCS score	−0.429	0.026	−0.456	0.045
Charlson index	0.556	0.003	0.546	0.014

SC, Spearman coefficient; NLR, neutrophil-to-lymphocyte ratio; PLR, platelet-to-lymphocyte ratio; SII, systemic immune–inflammation index; GPx3, glutathione peroxidase 3; Cu/Zn SOD, Cu/Zn superoxide dismutase; GCS, Glasgow Coma Scale.

## Data Availability

The data presented in this study are available on request from the corresponding author.

## Abbreviations

The following abbreviations are used in this manuscript:

AUC	Area under the curve
BM	Bacterial meningitis
BMS	Bacterial meningitis score
CI	Confidence interval
CNS	Central nervous system
CRP	C-reactive protein
CSF	Cerebrospinal fluid
Cu/Zn SOD	Cu/Zn superoxide dismutase
CV	Coefficient of variation
DNA	Deoxyribonucleic acid
GCS	Glasgow Coma Scale
GPx3	Glutathione peroxidase 3
GSH	Glutathione
GSSG	Glutathione disulfide
HIV	Human immunodeficiency virus
IL-6	Interleukin-6
mRS	Modified Rankin Scale
MAPK	Mitogen-activated protein kinase
NF-κB	Nuclear factor kappa-light-chain-enhancer of activated B cells
NLR	Neutrophil-to-lymphocyte ratio
OR	Odds ratio
OS	Oxidative stress
PCR	Polymerase chain reaction
PLR	Platelet-to-lymphocyte ratio
ROC	Receiver operating characteristic
ROS	Reactive oxygen species
SC	Spearman coefficient
SII	Systemic immune–inflammation index
TNF-α	Tumor necrosis factor α
VM	Viral meningitis
WBC	White blood cell
